# Clinical characterization, cardiovascular risk factor profile and cardiac strain analysis in a Uganda cancer population: The SATRACD study

**DOI:** 10.1371/journal.pone.0249717

**Published:** 2021-04-07

**Authors:** Wanzhu Zhang, Feriel Azibani, Emmy Okello, James Kayima, Isaac Sinabulya, Joseph Leeta, Victoria Walusansa, Jackson Orem, Karen Sliwa

**Affiliations:** 1 Hatter Institute of Cardiovascular Research in Africa, Cape Town, South Africa; 2 Uganda Heart Institute, Kampala, Uganda; 3 College of Health Science, Makerere University, Kampala, Uganda; 4 UMRS 942 Inserm, Paris, France; 5 Uganda Cancer Institute, Kampala, Uganda; Faculty of Medical Science - State University of Campinas, BRAZIL

## Abstract

**Background:**

The link between cancer and cardiovascular disease is firmly established. We sought to investigate the prevalence of cardiovascular disease (CVD) risk factors in Uganda cancer patients, their pre-chemotherapy left ventricular strain echocardiographic pattern and its associations with the CVD risk factors.

**Methods and results:**

Baseline pre-chemotherapy data of patients who were enrolled in the SATRACD study (a cancer cohort, who were planned for anthracycline therapy), were analyzed. The prevalence of cardiovascular risk factors and baseline strain echocardiographic images were assessed. Among the 355 patients who were recruited over a period of 15 months, 283 (79.7%) were female, with a mean age of 43 years. The types of cancer of the study patients included breast cancer (70.6%), lymphomas, sarcomas, leukemias and hepatocellular carcinoma. Hypertension was the most common comorbidity (27.0%). The prevalence of obesity was 12.1% and that of HIV was 18.3%. All patients had a normal left ventricular ejection fraction (LVEF). The mean global longitudinal strain (GLS) was -20.92 ±2.43%, with females having a significantly higher GLS than males (-21.09±2.42 vs -20.25±2.39, p = 0.008). Fifty-three patients (14.9%) had suboptimal GLS (absolute GLS≤18.00%), which was associated with obesity (POR = 3.07; 95% CI, 1.31–6.98; p = 0.003), alcohol use (POR = 1.94; 95% CI, 1.01–3.74; p = 0.044), long QTc interval in electrocardiogram (POR = 2.54; 95% CI, 1.06–5.74; p = 0.015,) and impaired left ventricular relaxation (POR = 2.24; 95% CI, 1.17–4.25; p = 0.007). On multivariable logistic regression analysis, obesity (POR = 2.95; 95% CI, 1.24–7.03; p = 0.014) was the only independent factor associated with suboptimal GLS.

**Conclusion:**

There is high prevalence and a unique pattern of cardiovascular risk factors in Uganda cancer patients. In cancer patients with cardiovascular risk conditions, there is reduction in GLS despite preserved LVEF. Longitudinal research is needed to study the predictive value of cardiovascular risk factors and baseline GLS for post chemotherapy cardiac dysfunction.

## Introduction

As in many low-middle income countries (LMICs), the growing burden of non-communicable diseases (NCDs) in Uganda is part of an epidemiologic shift catalyzed by demographic and nutritional transitions [1]. According to the World Health Organization published data in 2018, NCDs accounted for 33% of the mortality in Uganda. Cardiovascular disease (CVD) and cancer are the two leading causes of death from NCDs in the country [2].

The link between these two major NCDs is firmly established through shared risk factors, such as aging, tobacco use, physical inactivity, obesity, unhealthy diet, harmful use of alcohol and HIV status [1]. Cancer can directly or indirectly cause CVDs such as pericardial effusion, infiltrative cardiac disease and venous thromboembolic phenomenon. Moreover, CVDs can result from cancer therapy, e.g. cardiac dysfunction and hypertension due to certain chemotherapy regimens, coronary artery disease, valvular heart disease and pericardial disease due to chest radiotherapy [3]. The link between cancer and CVDs has created the need for joint care for cancer patients by cardiologists and oncologists.

The first step to improving the cardiac care of cancer patients is by knowing the CVD risks and baseline cardiac function of this patient population. Unfortunately, such data are lacking in most LMICs, including Uganda. This poses a great challenge to effective health sector planning and resource allocation.

The “Detecting Subclinical Anthracycline Related Cardiac Dysfunction In Low Income Country” (SATRACD) study is an ongoing cohort study of Ugandan cancer patients, which aims to determine the burden and risk factors for subclinical anthracycline therapy-related cardiac dysfunction. In this cohort, strain analysis by speckle tracking image is used as a major tool to detect subtle cardiac dysfunction resulting from anthracycline therapy. Strain echocardiography has been proven to be more sensitive in detecting subclinical cardiac dysfunction than conventional echocardiography parameters, and plays an important role in cardio-oncology practice [3]. We performed baseline data analysis from the SATRACD study, aiming to estimate the prevalence of CVD risk factors in Ugandan cancer patients, to describe the baseline strain pattern and to assess its associations with the CVD risk factors.

## Method

### Study design

This was a cross-sectional study from a cohort of cancer patients who were planned for anthracycline based chemotherapy.

### Study site and population

Patients were screened at the outpatient clinic of the Uganda Cancer Institute (UCI). Patient recruitment and data collection was done at the Uganda Heart Institute (UHI). Cancer patients who were planned for anthracycline therapy were referred to UHI for re-screening for eligibility. UCI is a specialized national referral oncology center in Kampala, Uganda. The center receives about 1,000 new cancer patients referred for chemotherapy annually. Currently, it has a bed capacity of about 100 beds with 8 specialist medical oncologists, 2 pharmacists, 10 medical officers and 50 trained oncology nurses. About 60% of patients attending UCI received anthracycline therapy. Uganda Heart Institute is a specialized provider of cardiovascular services and the only public referral facility for heart disease in Uganda. The institute handles over 20,000 patients annually, including over 95% of the adult patients and 58% of the cases among children in Uganda.

Three hundred and fifty-five (355) adult cancer patients, who were anthracycline naïve, were recruited consecutively between November 2018 and February 2020. The patient’s demographic data, cancer diagnosis, past medical history, symptoms, physical examinations, electrocardiography (ECG), echocardiography (ECHO) and laboratory data were collected. Because patients with impaired left ventricular (LV) function are not eligible for anthracycline chemotherapy, patients who had impaired LV systolic function (LV ejection fraction <50%) were excluded from the study. Patients who had poor quality 2-dimensional (2D) image or whose ECG showed left bundle branch block (LBBB) pattern were also excluded, as poor quality 2D image and LBBB ECG pattern would make the correct strain analysis impossible.

### Definition of the cardiovascular risk factors

Hypertension was defined as a systolic blood pressure (BP) ≥ 140 or diastolic BP ≥ 90 mmHg on any one of three measurements or self-reported current use of anti-hypertensives. Diabetes mellitus, HIV status, chronic kidney disease (CKD), smoking and alcohol consumption were defined based on the patient’s past medical record. Patient’s weight and height were measured for calculating body mass index (BMI) and body surface area (BSA). Under weight, normal weight, overweight and obesity were defined as BMI<18.5, 18.5≤ BMI≤25, 25< BMI <30 and BMI ≥30 respectively.

### Echocardiography protocol and equipment

Transthoracic ECHO images were acquired using the Vivid E9 (GE Healthcare) by two cardiologists. All ECHO images were analyzed by a single observer. LV ejection fraction (LVEF) was calculated using 2D method (Simpson biplane), from apical 4- and 2-chamber view, measured by “automatic EF” function with necessary manual adjustment. Stroke volume and cardiac index were calculated from both 2D method (apical 4- and 2-chamber views) and Doppler method (the time velocity integral of the pulsed wave Doppler signal of the LV outflow tract flow and area). Left atrial (LA) volume was measured using 2D biplane method (Simpson biplane) (from apical 4- and 2-chamber view) and indexed to BSA. Mitral annular plane systolic exertion (MAPSE) was measured by M-Mode obtained from apical 4-chamber view. Average of septal and lateral MAPSE was calculated. Tissue Doppler-derived indices was measured using the apical 4-chamber view. Peak systolic mitral annular velocities (S’) is calculated by averaging septal and lateral mitral annular velocities.

**Fig 1:**
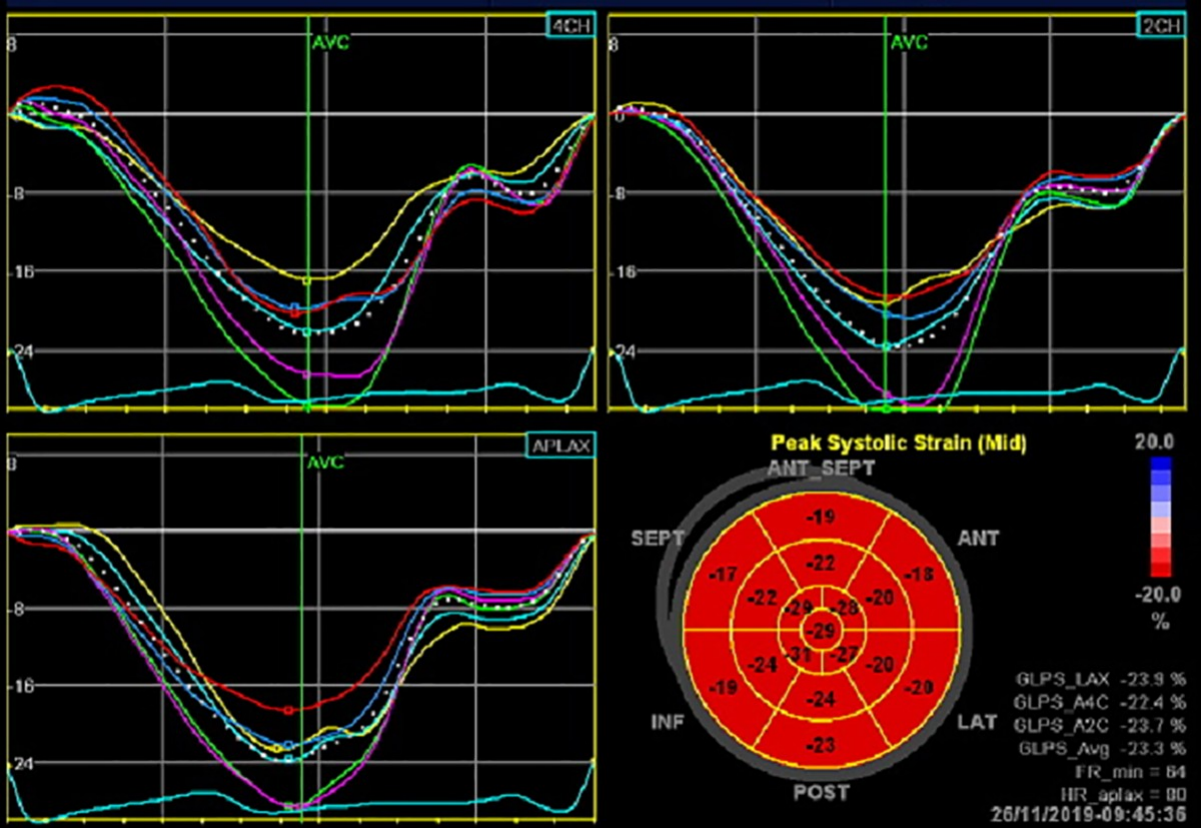
Global Longitudinal Strain (GLS) analysis.

For strain analysis, automatic speckle tracking strain analysis (“AFI” function) was used to measure LV global and regional longitudinal systolic strain from apical 4-, 2- and 3-chamber views. In the case of insufficient tracking, manual correction of the endocardial tracing was attempted. Regional longitudinal strain is demonstrated on a Bull’s eye diagram (Fig 1).

Automatic speckle tracking strain analysis (“AFI” function) was used to measure LV global and regional longitudinal systolic strain from apical 4-, 2- and 3-chamber views. Regional longitudinal strain was demonstrated on a Bull’s eye diagram.

Suboptimal GLS (low GLS) was defined as absolute GLS ≤ 18.00% [4].

To examine intra-observer reliability, we reassessed LV longitudinal strain assessments in a random sample of 10 patients. The intraclass correlation coefficients were 0.91, for LV global longitudinal strain (GLS) assessments.

### Ethical consideration

Ethical approvals were obtained from School of Medicine Ethics and Research Committee, College of Health Sciences Makerere University (REC REF 2018–081), Uganda National Council of Science and Technology (HS220ES) and Faculty of Health Sciences Human Research Ethics Committee, University of Cape Town (HREC 054/2020sa).

All participants were provided with a written copy of the consent form. Any and all questions that participants have regarding the research were addressed prior to signing of the consent form.

## Statistical analysis

We analyzed the data using Stata software (version 14). We summarized characteristics, laboratory results, ECG and ECHO assessments for patients enrolled before receiving anthracycline therapy. All continuous variables were expressed as a mean ± standard deviation (SD) and categorical variables as a percentage. Unpaired t test with Welch correction, paired t test and ANOVA were used to compare continuous variables. To assess the association between suboptimal (low) GLS and clinical characteristics, Prevalence Odds Ratio (POR) was calculated using Logistic regression. To control for the effect of the variables on the measures of association, all variables with a p-value of less than or equal to 0.2 were included in the multivariable model. A two-sided p-value <0.05 was considered statistically significant for all analyses.

## Results

**Fig 2:**
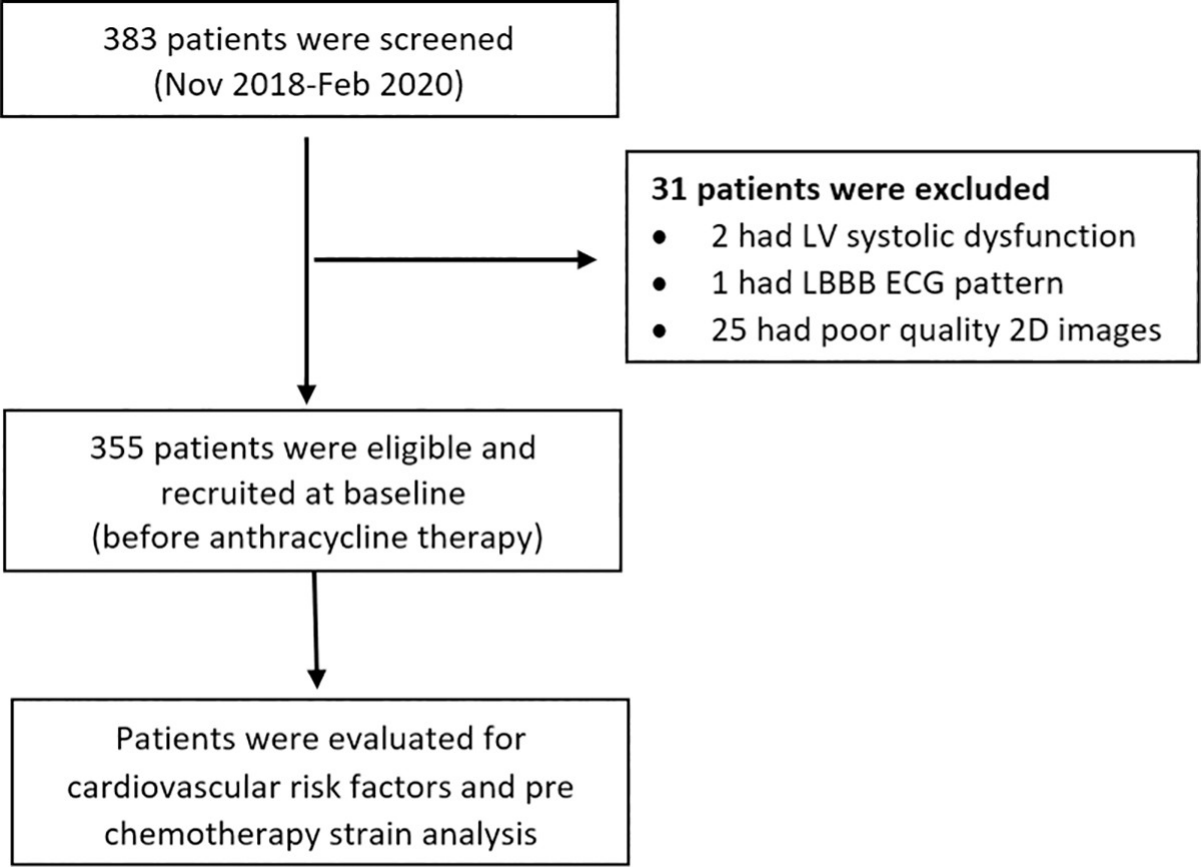
Patients’ flow chart.

A total of 383 patients were screened over a period of 15 months. Two patients were excluded due to LV systolic dysfunction, one patient was excluded due to LBBB ECG pattern, and twenty-five patients were excluded due to poor quality 2D images (Fig 2).

Among the 355 patients who were recruited, 283 (79.7%) were female, with a mean age of 43 years. Table 1 shows the clinical characteristics, laboratory and ECG data of the patients stratified by gender. Females had a significantly higher BMI, systolic BP and diastolic BP than males.

**Table 1:** Patient characteristics, laboratory and ECG data.

	Variables	Overall (N = 355)	Male (72, 20.3%)	Female (283, 79.7%)	*P-*value*
	Age(years)	43±12	41 ± 14	43 ± 11	0.14
Physical Examination					
	Body Mass Index (kg/m^2^)	24.7 ± 4.8	21.6±3.3	25.5 ± 4.8	<0.001
	Body Surface Area(m^2^)	1.7 ± 0.18	1.7 ± 0.16	1.7 ± 0.19	0.49
	Heart Rate (beats/min)	86 ± 16	88 ± 16	85 ± 17	0.19
	Systolic BP (mmHg)	129 ± 18	124 ± 14	131 ± 19	<0.001
	Diastolic BP (mmHg)	76 ± 12	74 ± 11	78 ± 12	0.008
	SaPO^2^ (%)	97 ± 2	96 ± 2	97 ± 2	0.15
Laboratory					
	Hemoglobin (g/dl)	12.6 ± 2.1	12.3 ± 2.8	12.7 ± 1.9	0.30
	eGFR (ml/min/1.73m^2^)	97.1 ± 2.1	99.8 ± 35.5	96.7 ± 30	0.67
	Troponin-I (ng/ml)	0.09 ± 0.12	0.12 ± 0.19	0.09 ± 0.09	0.14
Electrocardiography					
	PR interval (ms)	152 ± 22	152 ± 22	152 ± 21	0.96
	QTc interval (ms)	409 ± 28	405 ± 27	410 ± 28	0.19

*: *P* value derived from unpaired t-test with Welch correction

**Fig 3:**
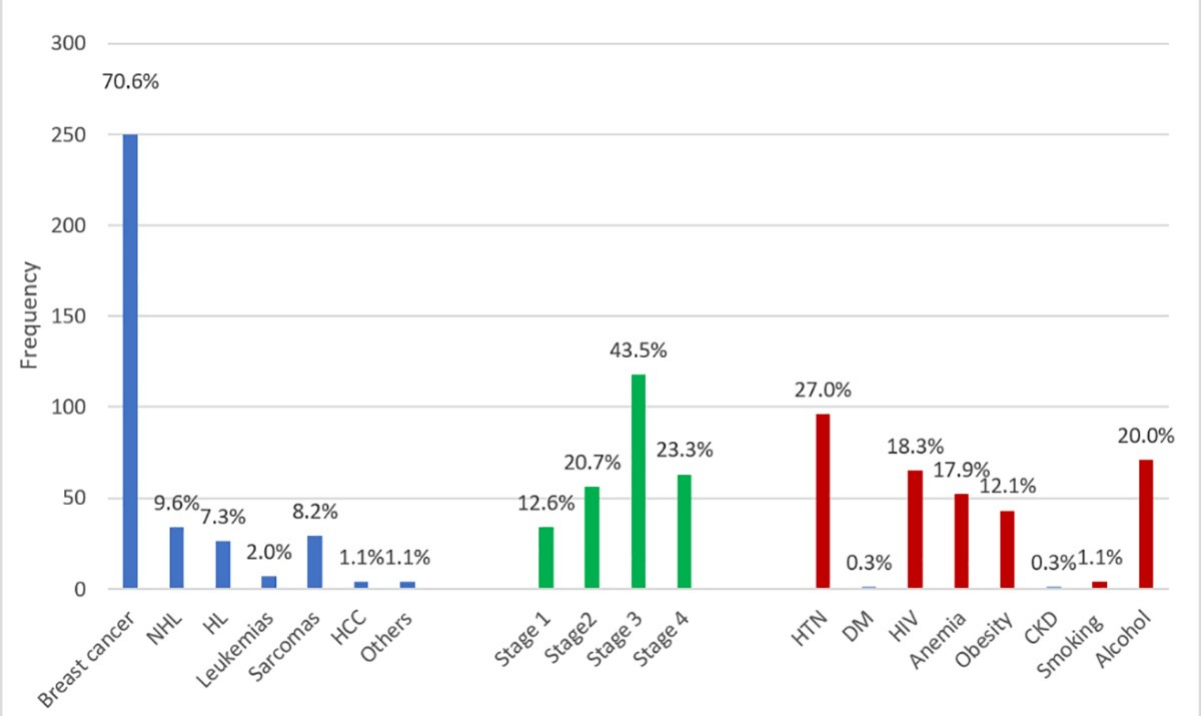
Cancer profile and cardiovascular risk factors. NHL: non-Hodgkin’s lymphoma, HL: Hodgkin’s lymphoma, HCC: hepatocellular carcinoma, HT: hypertension, DM: diabetes mellitus, CKD: chronic kidney disease.

Fig 3 shows the cancer type, staging and cardiovascular risk factors distribution of the study population. Breast cancer was the most commonly diagnosed cancer (250, 70.6%) in the group, followed by non-Hodgkin’s lymphoma (34, 9.6%), sarcomas (29, 8.2%), Hodgkin’s lymphoma (26, 7.3%), leukemia (7, 2.0%), hepatocellular carcinoma (4, 1.1%) and others (4, 1.1%). Most patients were found to have stage 3 (43.5%) and stage 4 (23.3%) diseases. Hypertension (27.0%) was the most common cardiovascular risk factor seen in the patients, followed by alcohol use (20.0%), HIV (18.3%), anemia (17.9%), obesity (12.1%), smoking (1.1%), Type 2 diabetes mellitus (0.3%) and CKD (0.3%).

Table 2 shows the conventional ECHO measurements and longitudinal strain ECHO measurements stratified by gender. For all the conventional ECHO parameters, male participants had significantly larger chamber sizes than females, except for LA volume. Male participants also had higher E’, stroke volume, cardiac index and lower E/E’ ratio than women. There was no difference in LV systolic function assessed by LVEF between male and female participants. The stroke volume and cardiac index assessed by two methods showed significant difference, with higher value obtained by Doppler method than 2D method.

**Table 2:** Echocardiography measurements.

Variables	Overall(N = 355)	Male (N = 72)	Female(N = 283)	*P-*valueǂ
LVDD (mm)	44.87 ± 4.85	47.22 ± 4.16	44.27 ± 4.84	<0.001
LVEDV (ml)	68.07 ± 18.18	81.43 ± 21.73	64.67 ± 1 5.45	<0.001
LVEDVI (ml/m^2^)	39.77 ± 9.96	46.98 ± 11.42	37.90 ± 8.64	<0.001
LVEF (%)	60.97 ± 5.66	60.93 ± 4.95	60.98 ± 5.83	0.94
MAPSE (mm)	14.49 ± 2.14	14.55 ± 2.17	14.48 ± 2.14	0.78
LAV (ml)	25.65± 9.74	27.39 ± 11.45	25.21 ± 9.23	0.12
LAVI (ml/m^2^)	14.93 ± 5.38	15.78 ± 6.37	14.72 ± 5.08	0.19
RVD (mm)	24.00 ± 2.94	26.00 ± 3.22	23.85 ± 2.71	<0.001
RAA (cm^2^)	10.90 ± 2.52	12.20 ± 2.75	10.58 ± 2.36	<0.001
TAPSE (mm^2^)	24.37 ± 3.98	24.37 ± 4.10	24.37 ± 3.96	0.99
E (cm/s)	81.49 ± 17.34	79.39 ± 18.75	82.04 ± 16.95	0.28
E/A	1.15 ± 0.37	1.24 ± 0.47	1.12 ± 0.34	0.062
E’ (cm/s)	12.22 ± 3.07	13.11 ± 3.43	12.00 ± 2.94	0.013
S ’ (cm/s)	9.62 ± 2.06	10.56 ± 2.08	9.38 ± 1.99	<0.001
E/E’	7.01 ± 2.04	6.32 ± 1.68	7.19 ± 2.09	<0.001
SV(2D) (ml)	41.65 ± 11.64	49.35 ± 14.2	39.70 ± 10.02	<0.001
SV(Doppler) (ml)	59.82 ± 15.91	65.64 ± 16.01	58.36 ± 15.58	<0.001
	*P*<0.001*			
CI(2D) (ml/L/m^2^)	2.05 ± 0.59	2.39 ± 0.66	1.96 ± 0.55	<0.001
CI(Doppler)(ml/L/m^2^)	2.87 ± 0.80	3.08 ± 0.82	2.81 ± 0.79	0.014
	***P*<0.001**^**#**^			
GLS (%)	-20.92 ± 2.43	-20.25 ± 2.39	-21.09 ± 2.42	0.009
LAX (%)	-20.74 ± 3.19	-20.13 ± 3.29	-20.90 ± 3.16	0.081
A4C (%)	-20.50 ± 2.91	-19.80 ± 2.32	-20.67 ± 3.01	0.011
A2C (%)	-21.50 ± 3.05	-20.70 ± 2.83	-21.70 ± 3.07	0.011
	***P* = 0.21**^**+**^			
Base (%)	-17.05 ± 2.82	-16.39 ± 2.99	-17.22 ± 2.75	0.034
Mid (%)	-20.10 ± 2.48	-19.53 ± 2.50	-20.25 ± 2.46	0.031
Apex (%)	-25.61 ± 3.88	-24.96 ± 3.52	-25.77 ± 3.96	0.091
	***P*<0.001**^**++**^			

LVDD: left ventricle diastolic diameter, LVEDV: left ventricle end diastolic volume, LVEDVI: left ventricle end diastolic volume index, LVEF: left ventricular ejection fraction, MAPSE: mitral annular plane systolic exertion, LAV: left atrium volume, RVD: right ventricular diameter, RAA: right atrium area, TAPSE: tricuspid annular plane systolic exertion, E: left ventricle inflow pulse wave Doppler E wave, E’: tissue Doppler mitral annular diastolic E’ velocity, S’: tissue Doppler mitral annular systolic velocity, SV(2D):2D stroke volume, SV(Doppler): Doppler stroke volume, CI(2D): 2D cardiac index, CI(Doppler): Doppler cardiac index, GLS: global longitudinal strain, LAX: apical long axis view A4C: apical 4 chamber view, A2C: apical 2 chamber view

ǂ: P- value derived from unpaired t test with Welch correction, comparing ECHO data between male and female patients.

*: P- value derived from Paired t test, comparing SV measured by 2D biplane method and Doppler method.

#: P- value derived from Paired t test, comparing CI measured by 2D biplane method and Doppler method

+: P- value derived from ANOVA, comparing segmental longitudinal strain at apical long axis view, apical 4 chamber view and apical 2 chamber view

++: P- value derived from ANOVA, comparing segmental longitudinal strain at the base, mid and apex of the left ventricle.

For strain analysis, the mean GLS for the study population was -20.92 ±2.43%, with males having significantly lower absolute GLS levels than females. Segmental strain analysis found that there was no difference among longitudinal strain in apical long axis view, apical 4 chamber view and apical 2 chamber view. There was significant longitudinal strain difference between the basal, mid and apical regions, with the basal region having the lowest absolute strain and apical having the highest absolute strain.

We defined suboptimal GLS (low GLS) as absolute GLS ≤ 18.00% [4]. There were 53(14.9%) patients were found to have low GLS. Table 3 shows the association analysis between patients’ clinical characteristics, cardiovascular risk factors, ECG and ECHO findings with low GLS. On bivariate analysis, we found low GLS associated with obesity, Alcohol use, QTc>440ms and impaired LV relaxation. After adjusting for patients’ sex, HIV status, alcohol use, overweight, obesity, QTc>440ms on ECG and impaired LV relaxation, obesity was the only independent factor associated with low GLS.

**Table 3:** Association of low GLS and variables.

Variables(N)	Low GLS (Total N = 53)	Bivariate models		Multivariable model	
	N(%)	POR(95%CI)	*P-*value	POR(95%CI)	*P-*value
Female(283)	37(13.07%)	0.53 (0.26–1.09)	0.052	0.54 (0.22–1.33)	0.17
Age>60 years(32)	4(12.50%)	0.80 (0.19–2.43)	0.69		
Hypertension(96)	14(14.58%)	1.11 (0.52–2.22)	0.77		
HIV(65)	14(21.54%)	1.77 (0.82–3.61)	0.098	1.08 (0.41–2.83)	0.87
Alcohol (71)	16(22.53)%	1.94 (1.01–3.74)	0.044	1.59 (0.93–2.71)	0.94
Tachycardia(66)	9(13.64)	1.67 (0.65–3.87)	0.21		
Normal BMI(184)	27(14.68%)	1			
Under Weight(21)	5(23.81%)	1.98 (0.52–6.34)	0.21		
Over Weight(102)	7(6.86%)	0.47 (0.16–1.17)	0.084	0.52 (0.21–1.32)	0.11
Obesity(43)	14(32.56%)	3.07 (1.31–6.98)	0.003	2.95 (1.24–7.03)	0.014
Hematological Malignancy(71)	14(19.72%)	1.43 (0.82–2.49)	0.21		
Stage 1 (34)	4(11.76%)	1			
Stage 2 (56)	11(19.64%)	1.83 (0.48–8.59)	0.33		
Stage 3 (117)	14(11.96%)	1.02 (0.29–4.57)	0.97		
Stage 4 (63)	13(20.63%)	1.95 (0.53–8.91)	0.27		
No Anemia(210)	30(14.28%)	1			
Mild Anemia (63)	12(19.05%)	1.44 (0.62–3.15)	0.33		
Mod- severe Anemia (18)	3(16.67%)	1.20 (0.21–4.62)	0.78		
Troponin>0.3ng/ml (6)	1(16.67%)	1.02 (0.02–9.40)	0.98		
QTc>440ms(40)	11(27.50%)	2.54 (1.06–5.74)	0.015	2.51 (0.95–6.62)	0.062
Impaired LV relaxation(84)	24(28.57%)	2.24 (1.17–4.25)	0.007	2.10 (0.97–4.58)	0.060

POR: Prevalence Odds Ratio

## Discussion

In this study, we assessed cardiovascular risk factors and cardiac function using strain imaging among cancer patients planned for anthracycline chemotherapy. Female patients and patients with breast cancer accounted for the majority of patients studied. In this group of cancer patients, we observed all the cardiovascular risk factors which are present in the general population, including hypertension (96, 27.0%), diabetes mellitus (1, 0.3%), obesity (43,12.1%), CKD (1, 0.3%), smoking (4, 1.1%), drinking alcohol (71, 20.0%), anemia (52,19.6%) and HIV (65,18.3%), among which hypertension is the most prevalent cardiovascular risk factor. These are the same findings reported in other cancer populations [5]. The latest nationwide NCDs risk factor survey in Uganda, with a younger mean age compared to our study population (31years verses 43years), revealed prevalence of hypertension, diabetes mellitus, obesity and alcohol use which were 26.5%, 1.4%,6.4% and 27.0% respectively [6, 7]. We found a similar prevalence of hypertension (27.0%) and alcohol use (20.0%) in our study patients. The lower prevalence of diabetes mellitus (0.3%) in our results could be due to a different study definition of the diabetes mellitus, whereby past medical records were used in our study rather than blood sugar testing. This difference may also suggest a potentially undiagnosed diabetes mellitus group in the community. More interestingly, obesity prevalence (12.1%) was strikingly higher in this cancer cohort than in the local population. Such result marks the fact that obesity promotes cancer, and breast cancer has been recognized as one of the obesity-associated cancers [8]. This also applies to another cancer promoter HIV, which accounts for 18.3% vs 6.5% in the study and general population [9] respectively. The most dramatic increase in cancers in Uganda has been noted due to the HIV epidemic in the country [10]. While obesity has been a traditional risk factor for CVD, recent evidence suggests that HIV-associated inflammation and immune activation are important mediators of cardiovascular risk [11].

Despite the burden of cardiovascular risk factors in this study group, they all have normal LV systolic function defined by LVEF at baseline. In order to detect subclinical cardiac dysfunction after anthracycline therapy, each patient was assessed with both conventional ECHO and speckle tracking image for strain analysis at baseline. Given that all patients had normal LVEF, do they all have normal strain values and what are the associations between their clinical characteristics and strain value? We therefore analyzed baseline ECHO data aiming to answer these questions.

Regarding the conventional ECHO assessment, all the patients had normal chamber sizes, with larger chamber sizes and higher stroke volume in males than females. There was no gender difference for LVEF. These findings correspond with the data generated from international study based on healthy population [12].

Longitudinal strain analysis by speckle tracking is a novel ECHO modality, which has been increasingly utilized in both clinical and research settings, including cardio-oncology practice. This is largely due to several advantages of this technique, such as higher sensitivity to detect cardiac dysfunction, good reproducibility and less volume dependent [3]. Our baseline pre-chemotherapy strain analysis showed the mean GLS value of -20.92 ±2.43%, with females having higher absolute GLS level than males. These results are in line with the findings in healthy populations [13] and similar cancer patients [14], with the same vendor-Vivid E9 (GE Healthcare). The longitudinal strain measured from apical long axis view, apical 4 chamber view and apical 2 chamber view did not show significant difference. However, the mean apical, mid and basal longitudinal strain showed stepward strain gradient, with apical region having the highest longitudinal strain. This pattern has been consistently observed in several studies [13, 15, 16]. Compared to the base, the apex is smaller and is subject to less wall stress, which may result in relatively higher longitudinal strain [17].

In this study, although all the patients had normal LVEF, 53(14.9%) patients were defined to have suboptimal absolute GLS (low GLS) of ≤18.00% [4]. We did not find association of low GLS with gender, age, hypertension, as found by other studies [18, 19]. Furthermore, factors such as HIV, anemia, tachycardia, cancer type and cancer stages were not associated with low GLS. Interestingly, obesity was the only independent factor associated with low GLS in our study. As we have discussed above, obesity, as a shared risk factor for cancer and cardiovascular disease, is not only found to have a high prevalence in this cancer cohort, but also related to low GLS. This has also been revealed by Wong C. Y and their colleges [20]. They studied 109 overweight or obese subjects and 33 referents (BMI <25 kg/m^2^) and found that the obese subjects (BMI >35 kg/m^2^) had reduced LV systolic and diastolic function, compared with referents (evidenced by lower average long-axis strain), whereas LVEF remained normal. It has been postulated that obesity leads to cardiac dysfunction through several direct and indirect mechanisms, including hemodynamic changes, myocardial fat accumulation, inflammatory cytokines, dyslipidemia and other comorbidities, e.g. diabetes mellitus, obstructive sleep apnea [21].

## Limitations

Our study is the first study to describe cardiovascular risk factors and pre-chemotherapy strain patterns in Ugandan cancer patients, although it has several limitations.

Our research patients, who were primarily selected for anthracycline therapy, were composed of certain cancer types. Therefore, the findings may not be generated for the whole cancer population in Uganda. Secondly, the ECHO observers were not blinded to patients’ status—this may lead to potential bias. However, automatic software was used for the strain analysis and 2D LVEF measurement, which we think can minimize the bias. Lastly, inter-observer variability was not assessed, despite that the ECHO images were acquired by two cardiologists. However, 97% of the patients’ ECHO images were recorded by one person and all the measurements were performed by a single observer.

## Conclusion

We found a high prevalence of cardiovascular risk factors in cancer patients, with hypertension being the most common, and a much higher prevalence of obesity and HIV than in the general population. Most patients had a normal strain pattern at the baseline. Among cancer patients, GLS is reduced, despite preserved LVEF, in the presence cardiovascular risk conditions such as obesity. Pre-existing suboptimal GLS and its associated risk factors could have important implications in cancer care. It is reasonable to assume that individuals with low GLS and associated risk factors at baseline will be more susceptible to chemotherapy related cardiac dysfunction and adverse outcomes. Therefore, longitudinal research is needed to study these hypotheses.

## Supporting information

S1 File (DOCX)
