# Supplementary material for: Clinical characterization, cardiovascular risk factor profile and cardiac strain analysis in a Uganda cancer population: The SATRACD study
Source: PLoS One. 2021 Apr 7;16(4):e0249717. doi: 10.1371/journal.pone.0249717 (PMC8026039; doi:10.1371/journal.pone.0249717)
Supplement: S1 File — (DOCX) [file pone.0249717.s001.docx]

Multiple liner regression analysis

| **GLS*** | ***Coefficients*** | ***P-value*** |
| --- | --- | --- |
|  |  |  |
| Age | 0.007 | 0.547 |
| Hemoglobin | -0.042 | 0.569 |
| Heart Rate | -0.013 | 0.184 |
| Systolic Blood Pressure | 0.028 | 0.013 |
| Diastolic Blood Pressure | -0.057 | 0.001 |
| Body Mass Index | -0.008 | 0.813 |
| QTc | -0.011 | 0.048 |

*: Dependent Variable

Normal distribution of the GLS value
